# The genome sequence of the oyster mushroom, *Pleurotus ostreatus *((Jacq.) P. Kummer, 1871)

**DOI:** 10.12688/wellcomeopenres.19578.1

**Published:** 2023-06-26

**Authors:** Richard Wright, Kieran Woof, Royal Botanic Gardens Kew Genome Acquisition Lab, Darwin Tree of Life Barcoding collective, Wellcome Sanger Institute Tree of Life programme, Wellcome Sanger Institute Scientific Operations: DNA Pipelines collective, Tree of Life Core Informatics collective, Darwin Tree of Life Consortium

**Affiliations:** 1 Royal Botanic Gardens Kew, Richmond, England, UK; 2 Cardiff University, Cardiff, Wales, UK

**Keywords:** Pleurotus ostreatus, oyster mushroom, genome sequence, chromosomal, Agaricales

## Abstract

We present a genome assembly from a *Pleurotus ostreatus* specimen (the oyster mushroom; Basidiomycota; Agaricomycetes; Agaricales; Pleurotaceae). The genome sequence is 40.6 megabases in span. Most of the assembly is scaffolded into 12 chromosomal pseudomolecules. Two mitochondrial genomes have been assembled, which are 73.1 and 9.3 kilobases in length.

## Species taxonomy

Eukaryota; Opisthokonta; Fungi; Dikarya; Basidiomycota; Agaricomycotina; Agaricomycetes; Agaricomycetidae; Agaricales; Pleurotineae; Pleurotaceae; *Pleurotus*; *Pleurotus ostreatus* ((Jacq.) P. Kummer, 1871) (NCBI:txid5322).

## Background

*Pleurotus ostreatus* (oyster mushroom) is a lamellate fungus that produces thin, fan-shaped, laterally-attached, fleshy sporocarps, with a dark grey, grey brown, bluish grey to yellow brown pileus, and an eccentric stem with strongly decurrent white lamellae. They grow in dense, imbricate clusters with each cap ranging in size from 50–150(–350) mm.

It has a monomitic hyphal system and the thin to thick-walled generative hyphae have clamps. Spores are subcylindrical-ellipsoid, smooth, hyaline, inamyloid, (7–)8–12.5 × (2–)3–4.5(5.5) μm, producing a white to pale lilac grey spore deposit ( Knudsen & Vesterholt, 2012).

In culture, the hyphae are hyaline, clamped and branched, with considerable aerial growth, and can produce subglobose to broadly ellipsoid thick-walled chlamydospores in aging cultures. In low nitrogen conditions, the hyphae can produce secretory cells that excrete powerful toxins that paralyse nematodes and cause shrinkage of their heads, before hyphal colonisation and digestion ( Soares *et al.*, 2018).

This species grows rapidly and aggressively in culture and is capable of producing sporocarps in short periods of time on a wide range of cellulose and lignin-based substrates. This, combined with desirable culinary characteristics and good keeping qualities, has led to *P. ostreatus* being one of the three most extensively cultivated fungi in the world ( Li *et al.*, 2020; Royse *et al.*, 2017).

*Pleurotus ostreatus* has a global distribution that includes all continents except Antarctica ( Li *et al.*, 2020). Throughout Europe it is known to cause a white-rot in the wood of a broad range of angiosperm trees, particularly *Fagus* and *Quercus*, either on standing trunks or larger fallen branches. In the UK, *P. ostreatus* is widespread and common wherever host trees are present. 

Oyster mushrooms have been shown to produce a diverse and valuable range of enzymes with biotechnological, therapeutic, and bioremediation uses ( Corrêa *et al.*, 2016; Knop *et al.*, 2015), and further discoveries, alongside improved taxonomic understanding of the cryptically diverse group will be aided by the production of this genome.

## Genome sequence report

**Figure 1.**
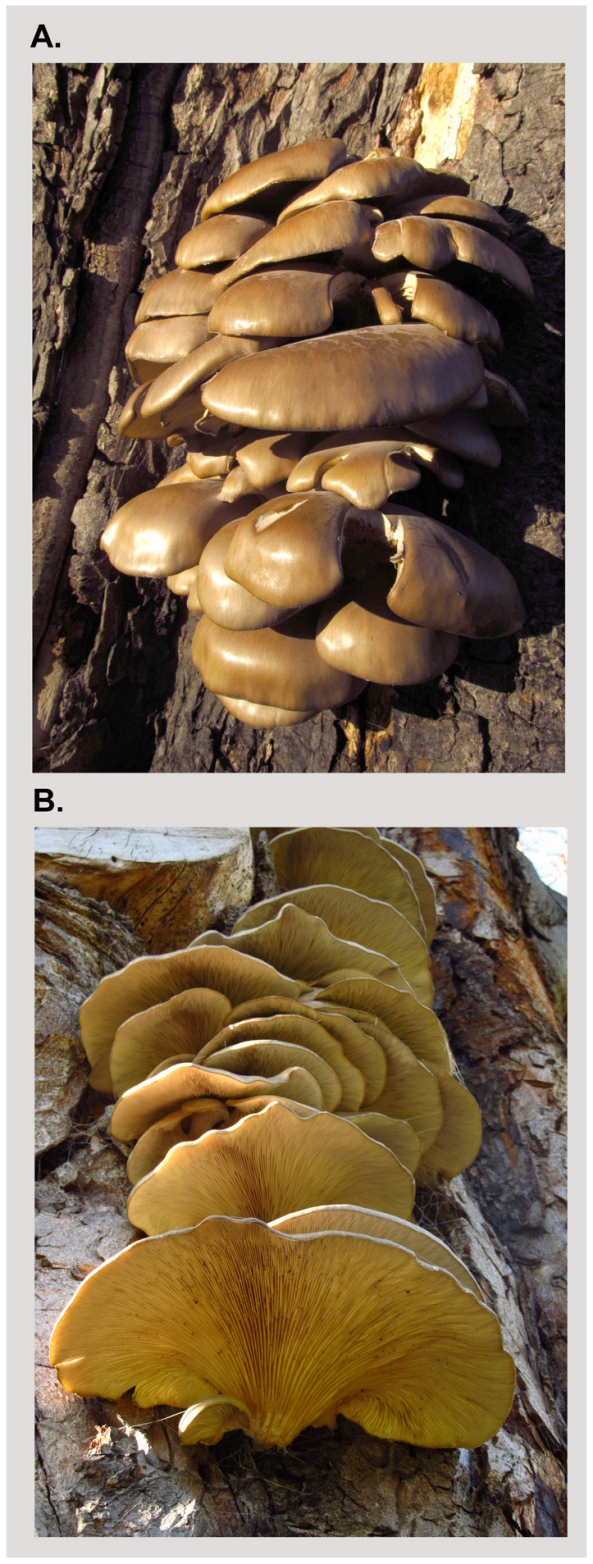
**A**. *Pleurotus* ostreatus sporocarps, growing on the trunk of *Aesculus hippocastanum*. **B**. The underside of the same sporocarps showing lamellae.

The genome was sequenced from a *Pleurotus ostreatus* specimen ( Figure 1) grown in a pure culture obtained from a single sporocarp collected at Eastville Park, Bristol, UK (latitude 51.48, longitude –2.56). A total of 57-fold coverage in Pacific Biosciences single-molecule HiFi long reads was generated. Primary assembly contigs were scaffolded with chromosome conformation Hi-C data. Manual assembly curation corrected four missing joins or mis-joins and removed one haplotypic duplication, increasing the scaffold count by three. 

**Figure 2.**
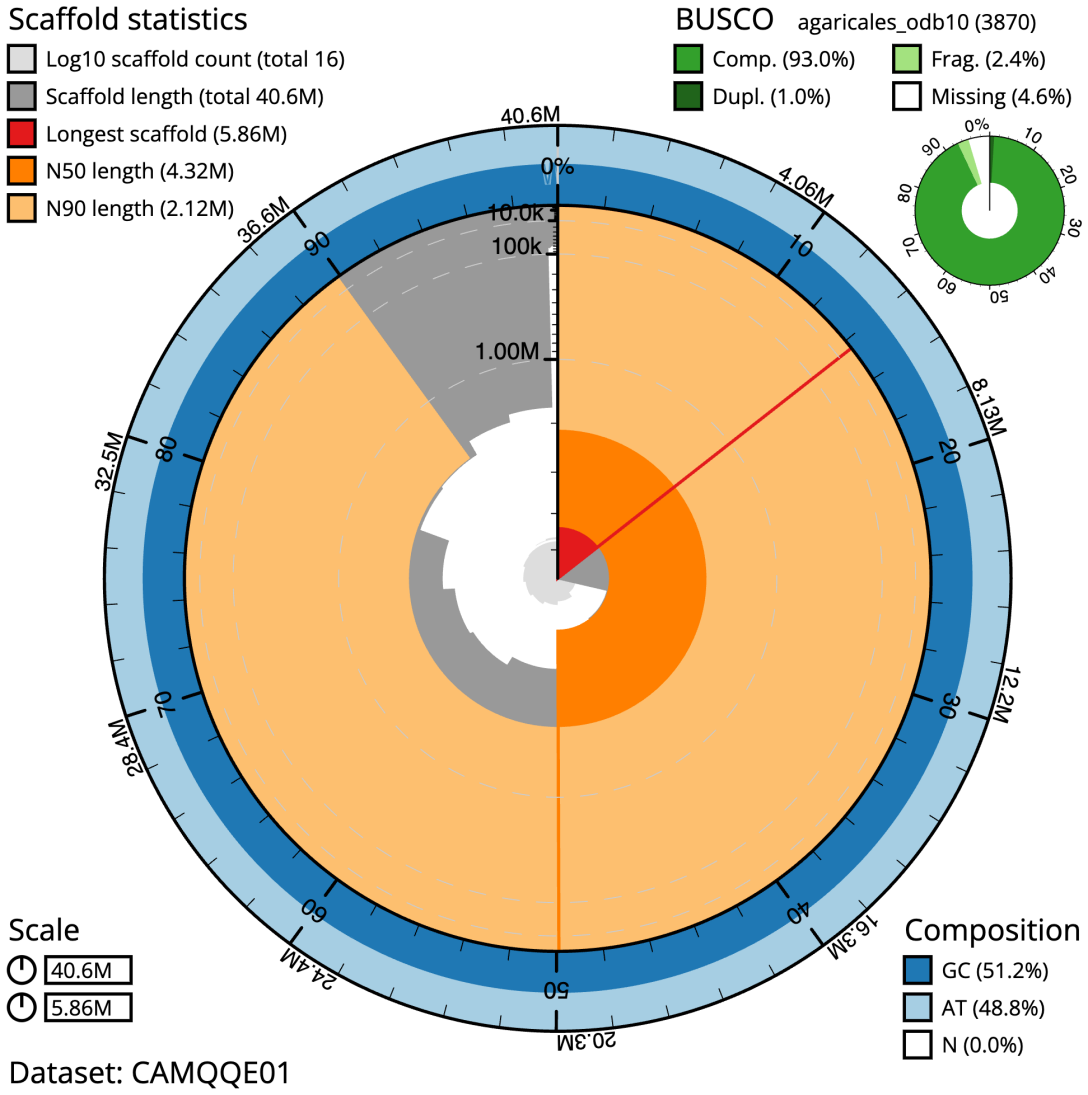
Genome assembly of *Pleurotus ostreatus*, gfPleOstr1.1: metrics. The BlobToolKit Snailplot shows N50 metrics and BUSCO gene completeness. The main plot is divided into 1,000 size-ordered bins around the circumference with each bin representing 0.1% of the 40,636,420 bp assembly. The distribution of scaffold lengths is shown in dark grey with the plot radius scaled to the longest scaffold present in the assembly (5,861,466 bp, shown in red). Orange and pale-orange arcs show the N50 and N90 scaffold lengths (4,316,464 and 2,120,766 bp), respectively. The pale grey spiral shows the cumulative scaffold count on a log scale with white scale lines showing successive orders of magnitude. The blue and pale-blue area around the outside of the plot shows the distribution of GC, AT and N percentages in the same bins as the inner plot. A summary of complete, fragmented, duplicated and missing BUSCO genes in the agaricales_odb10 set is shown in the top right. An interactive version of this figure is available at https://blobtoolkit.genomehubs.org/view/gfPleOstr1.1/dataset/CAMQQE01/snail.

The final assembly has a total length of 40.6 Mb in 14 sequence scaffolds with a scaffold N50 of 4.3 Mb ( Table 1). Most (99.79%) of the assembly sequence was assigned to 12 chromosomal-level scaffolds. Chromosome-scale scaffolds confirmed by the Hi-C data are named in order of size ( Figure 2– Figure 5; Table 2). There is a large heterozygous inversion on chromosome 5 from 569.3 kb to 2.176 Mb ( Figure 5). While not fully phased, the assembly deposited is of one haplotype. Contigs corresponding to the second haplotype have also been deposited. The mitochondrial genomes were also assembled and can be found as a contig within the multifasta file of the genome submission.

**Table 1.** Genome data for *Pleurotus ostreatus*, gfPleOstr1.1.

Project accession data		
Assembly identifier	gfPleOstr1.1	
Species	*Pleurotus ostreatus*	
Specimen	gfPleOstr1	
NCBI taxonomy ID	5322	
BioProject	PRJEB52213	
BioSample ID	SAMEA8562045	
Isolate information	gfPleOstr1; mycelium (DNA sequencing and HiC scaffolding)	
Assembly metrics *		*Benchmark*
Consensus quality (QV)	68.6	*≥ 50*
*k*-mer completeness	100%	*≥ 95%*
BUSCO **	C:93.0%[S:92.0%,D:1.0%], F:2.4%,M:4.6%,n:3,870	*C ≥ 95%*
Percentage of assembly mapped to chromosomes	99.79%	*≥ 95%*
Sex chromosomes	-	*localised homologous pairs*
Organelles	Two mitochondrial genomes assembled	*complete single alleles*
Raw data accessions		
PacificBiosciences SEQUEL II	ERR9630946, ERR9630947	
Hi-C Illumina	ERR9580486	
Genome assembly		
Assembly accession	GCA_947034855.1	
*Accession of alternate haplotype*	GCA_947034875.1	
Span (Mb)	40.6	
Number of contigs	17	
Contig N50 length (Mb)	4.3	
Number of scaffolds	14	
Scaffold N50 length (Mb)	4.3	
Longest scaffold (Mb)	5.9	

* Assembly metric benchmarks are adapted from column VGP-2020 of “Table 1: Proposed standards and metrics for defining genome assembly quality” from ( Rhie *et al.*, 2021). ** BUSCO scores based on the agaricales_odb10 BUSCO set using v5.3.2. C = complete [S = single copy, D = duplicated], F = fragmented, M = missing, n = number of orthologues in comparison. A full set of BUSCO scores is available at https://blobtoolkit.genomehubs.org/view/gfPleOstr1.1/dataset/CAMQQE01/busco.

**Figure 3.**
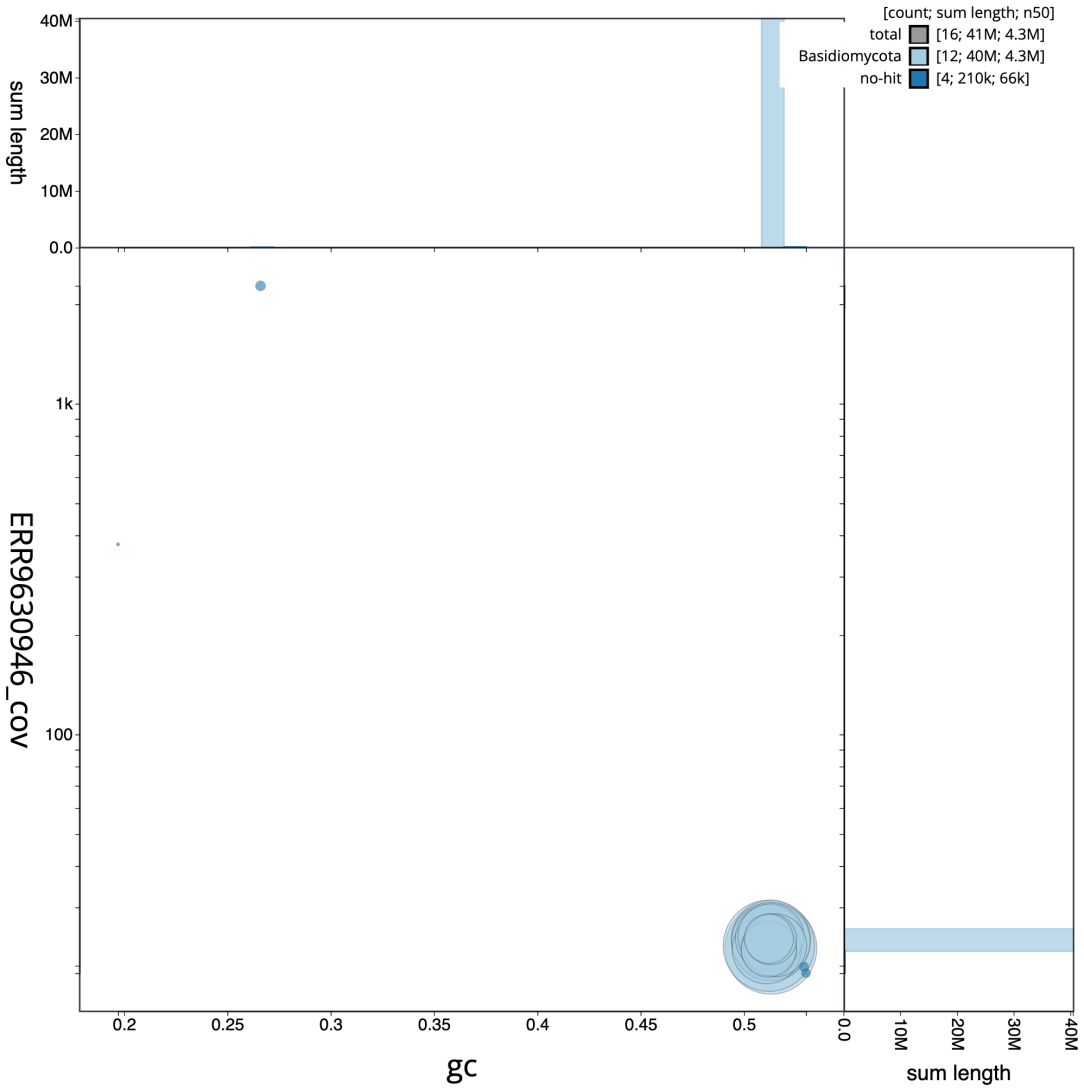
Genome assembly of *Pleurotus ostreatus*, gfPleOstr1.1: BlobToolKit GC-coverage plot. Scaffolds are coloured by phylum. Circles are sized in proportion to scaffold length. Histograms show the distribution of scaffold length sum along each axis. An interactive version of this figure is available at https://blobtoolkit.genomehubs.org/view/gfPleOstr1.1/dataset/CAMQQE01/blob.

**Figure 4.**
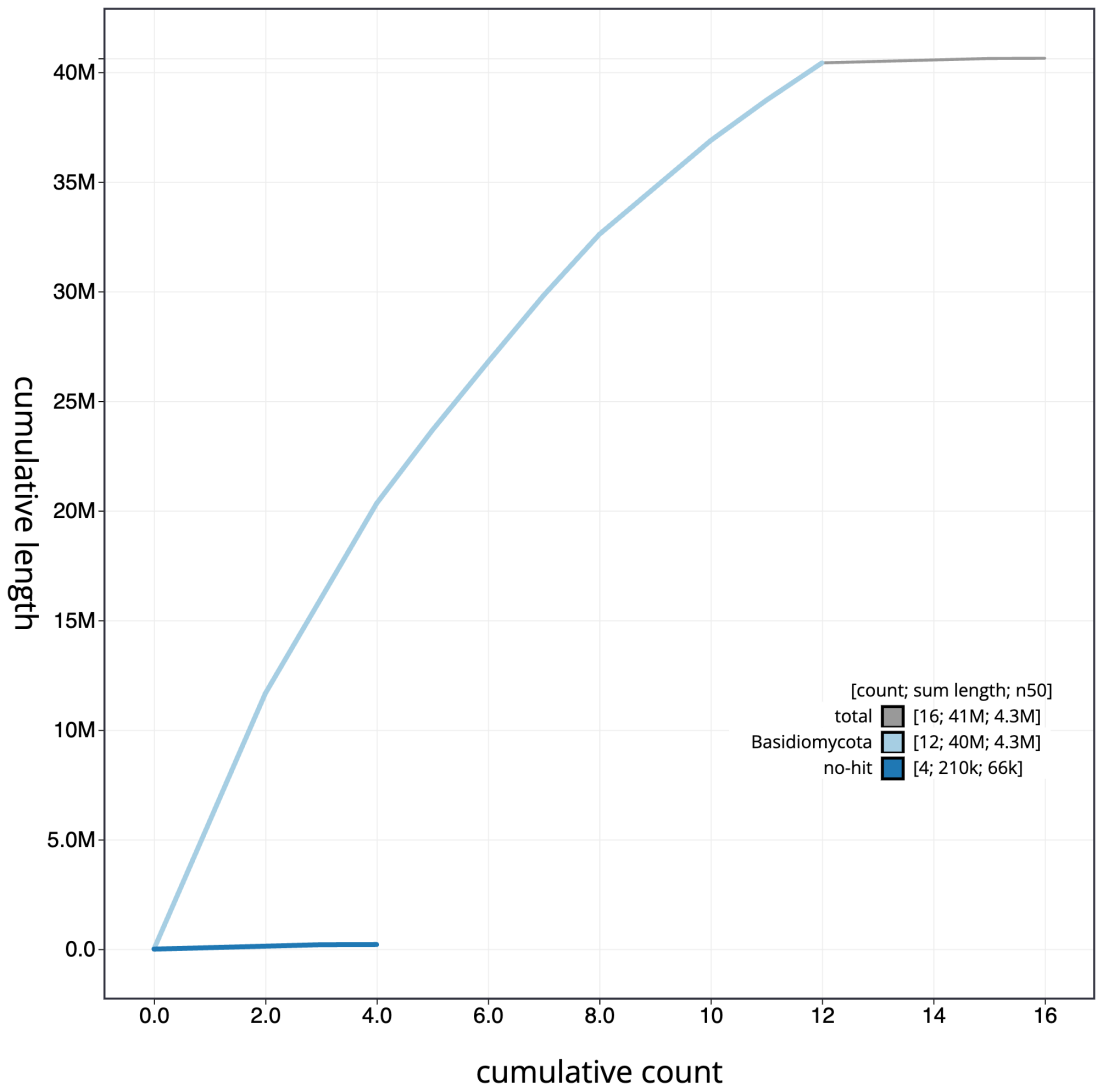
Genome assembly of *Pleurotus ostreatus*, gfPleOstr1.1: BlobToolKit cumulative sequence plot. The grey line shows cumulative length for all scaffolds. Coloured lines show cumulative lengths of scaffolds assigned to each phylum using the buscogenes taxrule. An interactive version of this figure is available at https://blobtoolkit.genomehubs.org/view/gfPleOstr1.1/dataset/CAMQQE01/cumulative.

**Figure 5.**
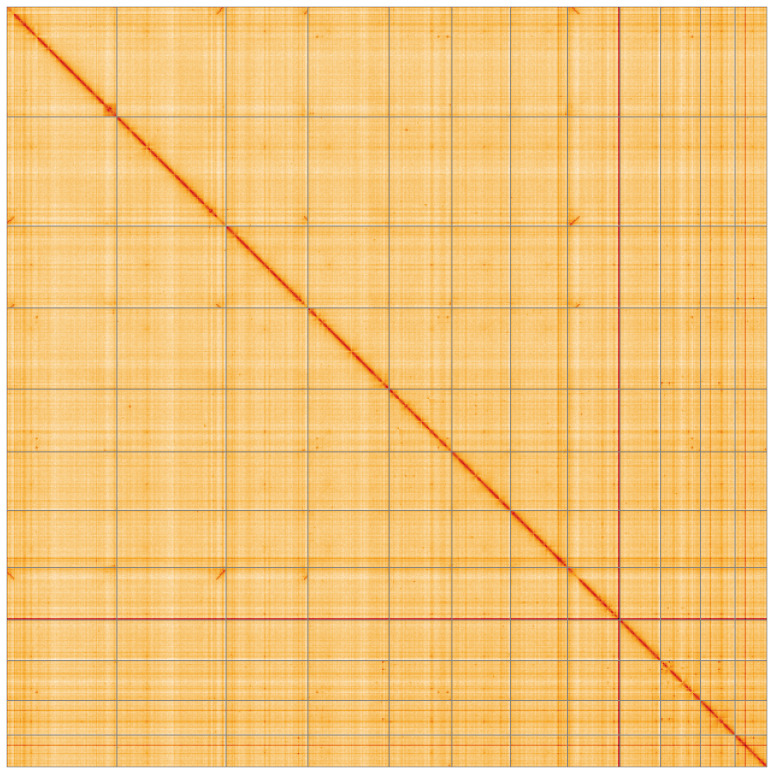
Genome assembly of *Pleurotus ostreatus*, gfPleOstr1.1: Hi-C contact map of the gfPleOstr1.1 assembly, visualised using HiGlass. Chromosomes are shown in order of size from left to right and top to bottom. An interactive version of this figure may be viewed at https://genome-note-higlass.tol.sanger.ac.uk/l/?d=RUr4Tdg_SNqA3C4JO5x1cA.

**Table 2.** Chromosomal pseudomolecules in the genome assembly of Pleurotus ostreatus, gfPleOstr1.

INSDC accession	Chromosome	Length (Mb)	GC%
OX344735.1	1	5.86	51.5
OX344736.1	2	5.8	51.0
OX344737.1	3	4.35	51.5
OX344738.1	4	4.32	51.5
OX344739.1	5	3.34	51.0
OX344740.1	6	3.11	51.0
OX344741.1	7	3.04	51.5
OX344742.1	8	2.78	51.5
OX344743.1	9	2.12	51.0
OX344744.1	10	2.16	51.0
OX344745.1	11	1.84	51.5
OX344746.1	12	1.7	51.0
OX344747.1	MT1	0.07	26.5
OX344748.1	MT2	0.01	19.5

The estimated Quality Value (QV) of the final assembly is 68.6 with *k*-mer completeness of 100%, and the assembly has a BUSCO v5.3.2 completeness of 93.0% (single = 92.0%, duplicated = 1.0%), using the agaricales_odb10 reference set ( *n* = 3,870).

Metadata for specimens, spectral estimates, sequencing runs, contaminants and pre-curation assembly statistics can be found at https://links.tol.sanger.ac.uk/species/5322.

## Methods

### Sample acquisition and nucleic acid extraction

A *Pleurotus ostreatus* specimen (gfPleOstr1) was collected at Eastville Park, Bristol, UK (latitude 51.48, longitude –2.56) by Richard Wright (RBGK, Cardiff University). The specimen was grown in pure culture obtained from a single sporocarp by Richard Wright. Culture handling and initial barcoding to confirm identity was carried out by Kieran Woof (RBGK) and samples taken from it were preserved on dry ice.

DNA was extracted at the Tree of Life laboratory, Wellcome Sanger Institute (WSI). The gfPleOstr1 sample was weighed and dissected on dry ice with tissue set aside for Hi-C sequencing. Tissue was cryogenically disrupted to a fine powder using a Covaris cryoPREP Automated Dry Pulveriser, receiving multiple impacts. High molecular weight (HMW) DNA was extracted using the Qiagen Plant MagAttract DNA extraction kit. HMW DNA was sheared into an average fragment size of 12–20 kb in a Megaruptor 3 system with speed setting 30. Sheared DNA was purified by solid-phase reversible immobilisation using AMPure PB beads with a 1.8X ratio of beads to sample to remove the shorter fragments and concentrate the DNA sample. The concentration of the sheared and purified DNA was assessed using a Nanodrop spectrophotometer and Qubit Fluorometer and Qubit dsDNA High Sensitivity Assay kit. Fragment size distribution was evaluated by running the sample on the FemtoPulse system.

### Sequencing

Pacific Biosciences HiFi circular consensus DNA sequencing libraries were constructed according to the manufacturers’ instructions. DNA sequencing was performed by the Scientific Operations core at the WSI on a Pacific Biosciences SEQUEL II (HiFi) instrument. Hi-C data were also generated from tissue of gfPleOstr1 using the Arimav2 kit and sequenced on the Illumina NovaSeq 6000 instrument.

### Genome assembly, curation and evaluation

Assembly was carried out with Hifiasm ( Cheng *et al.*, 2021) and haplotypic duplication was identified and removed with purge_dups ( Guan *et al.*, 2020). The assembly was then scaffolded with Hi-C data ( Rao *et al.*, 2014) using YaHS ( Zhou *et al.*, 2023). The assembly was checked for contamination as described previously ( Howe *et al.*, 2021). Manual curation was performed using HiGlass ( Kerpedjiev *et al.*, 2018) and Pretext ( Harry, 2022). The mitochondrial genome was assembled using MitoHiFi ( Uliano-Silva *et al.*, 2022), which runs MitoFinder ( Allio *et al.*, 2020) or MITOS ( Bernt *et al.*, 2013) and uses these annotations to select the final mitochondrial contig and to ensure the general quality of the sequence. The mitochondrial genome was assembled using MitoHiFi. The mitochondrial linear plasmid MT2 was assembled using MBG ( Rautiainen & Marschall, 2021).

A Hi-C map for the final assembly was produced using bwa-mem2 ( Vasimuddin *et al.*, 2019) in the Cooler file format ( Abdennur & Mirny, 2020). To assess the assembly metrics, the *k*-mer completeness and QV consensus quality values were calculated in Merqury ( Rhie *et al.*, 2020). This work was done using Nextflow ( Di Tommaso *et al.*, 2017) DSL2 pipelines “sanger-tol/readmapping” ( Surana *et al.*, 2023a) and “sanger-tol/genomenote” ( Surana *et al.*, 2023b). The genome was analysed within the BlobToolKit environment ( Challis *et al.*, 2020) and BUSCO scores ( Manni *et al.*, 2021; Simão *et al.*, 2015) were calculated.

Table 3 contains a list of relevant software tool versions and sources.

**Table 3.** Software tools: versions and sources.

Software tool	Version	Source
BlobToolKit	4.0.7	https://github.com/blobtoolkit/blobtoolkit
BUSCO	5.3.2	https://gitlab.com/ezlab/busco
Hifiasm	0.16.1-r375	https://github.com/chhylp123/hifiasm
HiGlass	1.11.6	https://github.com/higlass/higlass
MBG	-	https://github.com/maickrau/MBG
Merqury	MerquryFK	https://github.com/thegenemyers/MERQURY.FK
MitoHiFi	2	https://github.com/marcelauliano/MitoHiFi
PretextView	0.2	https://github.com/wtsi-hpag/PretextView
purge_dups	1.2.3	https://github.com/dfguan/purge_dups
sanger-tol/genomenote	v1.0	https://github.com/sanger-tol/genomenote
sanger-tol/readmapping	1.1.0	https://github.com/sanger-tol/readmapping/tree/1.1.0
YaHS	yahs-1.1.91eebc2	https://github.com/c-zhou/yahs

### Wellcome Sanger Institute – Legal and Governance

The materials that have contributed to this genome note have been supplied by a Darwin Tree of Life Partner. The submission of materials by a Darwin Tree of Life Partner is subject to the **‘Darwin Tree of Life Project Sampling Code of Practice’**, which can be found in full on the Darwin Tree of Life website here. By agreeing with and signing up to the Sampling Code of Practice, the Darwin Tree of Life Partner agrees they will meet the legal and ethical requirements and standards set out within this document in respect of all samples acquired for, and supplied to, the Darwin Tree of Life Project.

Further, the Wellcome Sanger Institute employs a process whereby due diligence is carried out proportionate to the nature of the materials themselves, and the circumstances under which they have been/are to be collected and provided for use. The purpose of this is to address and mitigate any potential legal and/or ethical implications of receipt and use of the materials as part of the research project, and to ensure that in doing so we align with best practice wherever possible. The overarching areas of consideration are:

• Ethical review of provenance and sourcing of the material

• Legality of collection, transfer and use (national and international) 

Each transfer of samples is further undertaken according to a Research Collaboration Agreement or Material Transfer Agreement entered into by the Darwin Tree of Life Partner, Genome Research Limited (operating as the Wellcome Sanger Institute), and in some circumstances other Darwin Tree of Life collaborators.

## Data Availability

European Nucleotide Archive: *Pleurotus ostreatus* (oyster mushroom). Accession number PRJEB52213; https://identifiers.org/ena.embl/PRJEB52213. ( Wellcome Sanger Institute, 2022) The genome sequence is released openly for reuse. The *Pleurotus ostreatus* genome sequencing initiative is part of the Darwin Tree of Life (DToL) project. All raw sequence data and the assembly have been deposited in INSDC databases. The genome will be annotated using available RNA-Seq data and presented through the Ensembl pipeline at the European Bioinformatics Institute. Raw data and assembly accession identifiers are reported in Table 1.

## Author information

Members of the Royal Botanic Gardens Kew Genome Acquisition Lab are listed here: https://doi.org/10.5281/zenodo.4786680.

Members of the Darwin Tree of Life Barcoding collective are listed here: https://doi.org/10.5281/zenodo.4893703.

Members of the Wellcome Sanger Institute Tree of Life programme are listed here: https://doi.org/10.5281/zenodo.4783585.

Members of Wellcome Sanger Institute Scientific Operations: DNA Pipelines collective are listed here: https://doi.org/10.5281/zenodo.4790455.

Members of the Tree of Life Core Informatics collective are listed here: https://doi.org/10.5281/zenodo.5013541.

Members of the Darwin Tree of Life Consortium are listed here: https://doi.org/10.5281/zenodo.4783558.
