# Supplementary material for: The genome sequence of the oyster mushroom, Pleurotus ostreatus ((Jacq.) P. Kummer, 1871)
Source: Wellcome Open Res. 2023 Jun 26;8:277. [Version 1] doi: 10.12688/wellcomeopenres.19578.1 (PMC11112307; doi:10.12688/wellcomeopenres.19578.1)
Supplement: Peer review history

# Reviewer report: Reviewer response for version 1

**DOI:** 10.21956/wellcomeopenres.21688.r83901

**Published:** 2024-05-22

**Authors:** Erich M. Schwarz

**Affiliations:** 1 Cornell University, Ithaca, New York, USA

This description of a *Pleurotus ostreatus* genome assembly is concise, complete, and credible.  The genome has been properly archived in GenBank, the methods described are appropriate, and both the raw data and the software tools are rigorously described with URLs for data or code files.

 I observe only one error (which a previous reviewer pointed out a year ago!).  In the Methods, there is a second copy of the sentence "The mitochondrial genome was assembled using MitoHiFi.", immediately before the sentence "The mitochondrial linear plasmid MT2 was assembled using MBG...".  This second sentence about using MitoHiFi is redundant and should be deleted.

 I also note that in their Data Availability section, the authors promise, "The genome will be annotated using available RNA-Seq data and presented through the Ensembl pipeline at the European Bioinformatics Institute."  There may exist RNA-seq data for *Pleurotus ostreatus*, but the authors do not provide a citation or web link for them, and it is not clear if this annotation will ever happen (I did not find any such annotation in Ensembl on 21 May 2025).  It would be good if such RNA-seq data really existed and was used to provide protein-coding gene predictions for this high-quality fungal genome.

Are sufficient details of methods and materials provided to allow replication by others?

Yes

Is the rationale for creating the dataset(s) clearly described?

Yes

Are the datasets clearly presented in a useable and accessible format?

Yes

Are the protocols appropriate and is the work technically sound?

Yes

Reviewer Expertise:

Nematode genomics and transcriptomics.

I confirm that I have read this submission and believe that I have an appropriate level of expertise to confirm that it is of an acceptable scientific standard.

# Reviewer report: Reviewer response for version 1

**DOI:** 10.21956/wellcomeopenres.21688.r61548

**Published:** 2023-07-14

**Authors:** Alexandra Dallaire

**Affiliations:** 1 Department of Biochemistry, University of Cambridge, Cambridge, England, UK

The manuscript by Wright and Woof describe the assembly and curation of high quality nuclear and mitochondrial genomes of a *Pleurotus ostreatus* specimen. The experiments were carefully performed and I have no concern regarding their validity.

 Some aspects of the manuscript can be clarified.

 Specific points to be addressed:

1. The authors identify two pseudomolecules as mitochondrial genomes and name them MT1 and MT2. These scaffolds are of two different lengths (73Kb and 9Kb respectively). The smallest mitochondrial molecule MT2 is later on referred to as a mitochondrial plasmid. Mitochondrial plasmids have been described in plants, fungi, and ciliates, and differ from core mitochondrial genomes in that they are linear rather than circular. Since the mitochondrial genome of this species is proposed to be multipartite and of different architectures (circular MT1 and linear MT2), these distinctions could be clarified throughout the abstract and text.
2. Methods [Genome assembly, curation and evaluation]. The sentence “The mitochondrial genome was assembled using MitoHiFi.” appears twice.

Are sufficient details of methods and materials provided to allow replication by others?

Yes

Is the rationale for creating the dataset(s) clearly described?

Yes

Are the datasets clearly presented in a useable and accessible format?

Yes

Are the protocols appropriate and is the work technically sound?

Yes

Reviewer Expertise:

Genomics, epigenetics, fungal biology

I confirm that I have read this submission and believe that I have an appropriate level of expertise to confirm that it is of an acceptable scientific standard.
